# Phosphorylated heat shock protein 27 as a potential biomarker to predict the role of chemotherapy-induced autophagy in osteosarcoma response to therapy

**DOI:** 10.18632/oncotarget.20308

**Published:** 2017-08-17

**Authors:** Janice M. Santiago-O’Farrill, Eugenie S. Kleinerman, Mario G. Hollomon, Andrew Livingston, Wei-Lien Wang, Jen-Wei Tsai, Nancy B. Gordon

**Affiliations:** ^1^ Division of Pediatrics, The University of Texas MD Anderson Cancer Center, Houston, Texas, USA; ^2^ Department of Biology, Texas Southern University, Houston, Texas, USA; ^3^ Division of Cancer Medicine, The University of Texas MD Anderson Cancer Center, Houston, Texas, USA; ^4^ Department of Translational Molecular Pathology, The University of Texas MD Anderson Cancer Center, Houston, Texas, USA

**Keywords:** osteosarcoma, autophagy, gemcitabine, hydroxychloroquine, Beclin

## Abstract

Autophagy is a catabolic process involved in cellular homeostasis. Autophagy is increased above homeostatic levels by chemotherapy, and this can either promote or inhibit tumor growth. We previously demonstrated that aerosol gemcitabine (GCB) has a therapeutic effect against osteosarcoma (OS) lung metastases. However, some tumor cells failed to respond to the treatment and persisted as isolated lung metastasis. Here, we examined the mechanisms underlying the dual role of chemotherapy-induced autophagy in OS and sought to identify biomarkers to predict OS response to treatment. In this study, we demonstrate that treatment of various OS cells with GCB induced autophagy. We also showed that GCB reduces the phosphorylation of AKT, mTOR and p70S6K and that GCB-induced autophagy in OS can lead to either cell survival or cell death. Blocking autophagy enhanced the sensitivity of LM7 OS cells and decreased the sensitivity of CCH-OS-D and K7M3 OS cells to GCB. Using a kinase array, we also demonstrated that differences in the phosphorylated heat shock protein 27 (p-HSP27) expression in the various OS cell lines after treatment with GCB, correlates to whether chemotherapy-induced autophagy will lead to increase or decrease OS cells sensitivity to therapy. Increased p-HSP27 was associated with increased sensitivity to anticancer drug treatment when autophagy is inhibited. The results of this study reveal a dual role of autophagy in OS cells sensitivity to chemotherapy and suggest that p-HSP27 could represent a predictive biomarker of whether combination therapy with autophagy modulators and chemotherapeutic drugs will be beneficial for OS patients.

## INTRODUCTION

Osteosarcoma (OS) is the most common primary malignant bone tumor. Incidence is highest in the adolescent and young adult population [1]. Despite advances in the chemotherapy regimen used to treat OS, the 5-year overall survival rates for patients with OS have remained unchanged at 65-70% for the past 20 years. Disease relapse usually occurs in the lungs. Although aggressive multidisciplinary treatment with perioperative chemotherapy and surgery can have a therapeutic benefit in the primary tumor, pulmonary metastases remain, and these constitute the most common cause of death in patients with OS. The 5-year overall survival rate is only 30-35% in patients with metastatic disease at diagnosis [2], [3]. Treatment of pulmonary metastatic disease with systemic therapy has been only modestly effective and poses a clinical challenge, highlighting the need for new therapeutic strategies to the currently available treatment regimens.

Gemcitabine (GCB), a nucleoside analogue that inhibits DNA synthesis and induces apoptosis, has shown activity against many solid tumors, including OS [4, 5]. We previously demonstrated that aerosol GCB had a significant therapeutic effect against OS lung metastases in various OS mouse models, including the human LM7 and the murine DLM8 and K7M3 models [2, 6]. However, GCB therapeutic efficacy is limited, possibly in part by acquired tumor cell resistance to chemotherapy, as demonstrated by the presence of small isolated tumor nodules at the end of therapy that lead to relapse and death. Better understanding of the molecular mechanisms involved in OS response or resistance to chemotherapy is needed to improve the therapeutic effect of the current chemotherapy regimens and to increase survival rates.

Autophagy has been identified as one of the molecular mechanisms implicated in tumor cell resistance to chemotherapy. Autophagy is defined as a catabolic process by which cells maintain homeostasis [7]. It involves the sequestration of cytoplasmic material, long-lived proteins, and damaged organelles within a double membrane structure, called an autophagosome. The autophagosome then fuses with lysosomes, forming an autophagolysosome in which the sequestered material is degraded and used as substrates to generate energy [8]. Autophagy levels can be increased in cancer cells by stressful conditions such as starvation, hypoxia, and chemotherapy. This increased autophagy may serve as a cell survival mechanism, providing the cells with amino acids and fatty acids as a source of energy [8]. However, evidence suggests that excessive autophagy can also lead to cell death [9]. Therefore, autophagy has emerged as a significant mechanism involved in the response of cancer cells to chemotherapy [10, 11].

We have previously demonstrated that inhibition of camptothecin (CPT)-induced autophagy in DLM8 and K7M3 OS cells, decreased CPT-induced cytotoxicity in DLM8 cells and increased CPT-induced cytotoxicity in K7M3 cells, confirming that autophagy can both promote and inhibit antitumor drug response [12]. However, what determines this dual role is unknown. The p53 status was shown to determine the role of autophagy in pancreatic tumor development and in the response of lung cancer cells to radiation [13–15]. However, in OS, p53 status does not affect autophagy or response to autophagy inhibition [12]. Therefore, further understanding of the transcriptional regulators that determine whether induction of autophagy leads to tumor cell survival or death is needed to further develop more effective combination therapies. Current clinical trials are evaluating the use of autophagy inhibitors alone or in combination as adjuvant therapy for the treatment of multiple tumor types, including glioblastoma, melanoma, myeloma, and renal cell carcinoma [16–18].

In the present study, we sought to elucidate the mechanisms involved in the dual role of autophagy in the response of OS to chemotherapy. Here, we demonstrated that GCB induces autophagy in various OS cell lines both in vitro and in vivo, and that GCB-induced autophagy leads to downregulation of the AKT/mTOR signaling pathway. Furthermore, inhibition of autophagy by hydroxychloroquine (HCQ) or shRNA targeting BECN or Atg5 led to increased sensitivity to GCB in LM7 OS cells and decreased sensitivity to GCB in the CCH-OS-D and K7M3 cells, confirming the dual role of autophagy in OS. Currently, there are no biomarkers to determine whether chemotherapy-induced autophagy will lead to cell survival or death. Here, we evaluated heat shock protein 27 (HSP27) as a potential biomarker to predict the role of autophagy in OS response to chemotherapy.

## RESULTS

### GCB has cytotoxic effects within various OS cell lines

**Figure 1:**
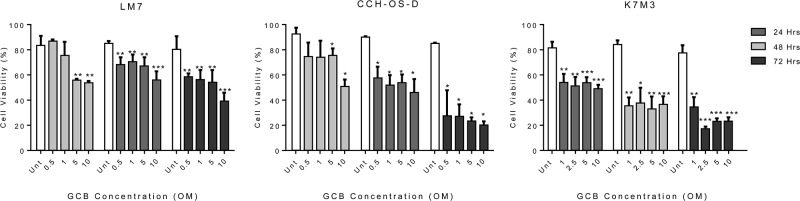
Gemcitabine (GCB) reduces cell viability in human osteosarcoma cells LM7, CCH-OS-D, and K7M3 cells were treated with GCB as indicated in figure for 24, 48, and 72 hours. Cell viability was determined by Trypan blue exclusion assay. Means ± standard deviation of three independent experiments are shown. *p < 0.05 compared with control.

Three metastatic OS cell lines, LM7, CCH-OS-D, and K7M3, demonstrated sensitivity to GCB at various drug concentrations (0.5-10μM) and time points (24, 48, or 72 hours). Trypan blue exclusion assay was used to determine the cell viability (Figure 1). Treatment with GCB resulted in decreased cell viability in all the OS cell lines tested. Viability of LM7 cells was significantly decreased when cells were exposed to the highest doses of GCB (5 and 10μM) over a period of 24 hours whereas no significant decrease in cell viability was observed at the lower GCB doses. However, after 48 hours, LM7 cells viability was significantly decreased with all the GCB doses tested. Similarly, for the CCH-OS-D cells, a significant decrease in cell viability was observed after 24 hours of treatment with GCB at the 5 and 10μM doses but, at 48 hours, a significant decrease in cell viability was observed not only with the highest doses but with all the doses tested. As shown in Figure 1, a significant decrease in cell viability was also observed in the murine K7M3 cells as early as 24 hours following treatment at the 1uM dose concentration with only further decrease in viability 48 hours post-treatment with the rest of the doses. By 72 hours, sensitivity to GCB varied among the three OS cell lines at the 10μM GCB dose. Cell viability was higher in the LM7 cells compared to the CCH-OS-D and K7M3 cells following GCB treatment, suggesting that the LM7 cells are less sensitive than the CCH-OS-D and K7M3 cells to the cytotoxic effect of GCB (Figure 1).

### GCB induces autophagy in vitro in OS

Despite the significant cytotoxic effect of GCB against the three OS cell lines tested, a population of cells remained viable suggesting the induction of a possible alternative mechanism able to enhance tumor cell survival and eventually limit GCB therapeutic efficacy. We therefore sought to determine whether GCB-induced autophagy in LM7, CCH-OS-D, and K7M3 OS cells may contribute to the survival of this population of tumor cells.

**Figure 2:**
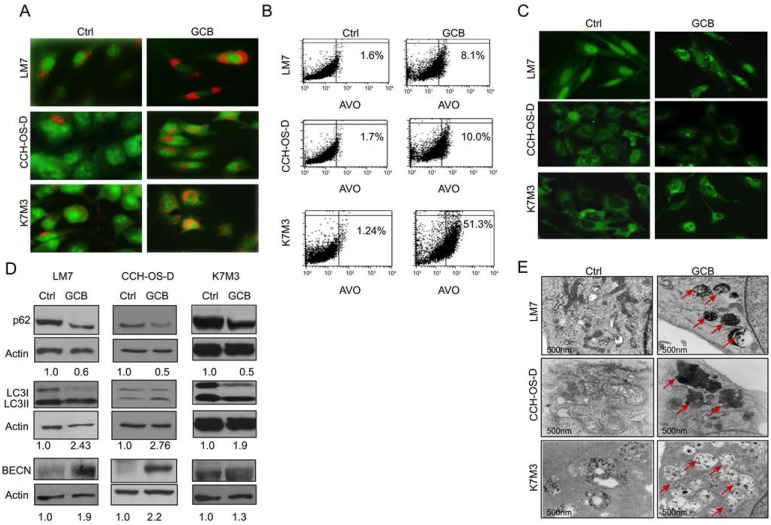
Treatment with gemcitabine (GCB) induces autophagy in osteosarcoma cells **A.** Following 48 hours of treatment with 1μM GCB, LM7, CCH-OS-D, and K7M3 cells were stained with the lysosomotropic agent acridine orange (1 μg/ml) for 15 minutes and then visualized under a confocal microscope. Treatment with GCB led to the formation of acidic vesicular organelles (AVOs –red/orange), as shown in the representative images. **B.** Flow cytometry was used to measure and quantify AVO formation in untreated (left) and treated (right) osteosarcoma cells. **C.** Cells were treated with 1μM GCB for 48 hours, and after fixation, cells were stained with anti-LC3 antibody and analyzed by fluorescence confocal microscopy. GCB increases the number of green punctae in the treated group as compared to the untreated. **D.** Expression of BECN, LC3, and p62 was analyzed using Western blot analysis. Cells were treated with GCB for 48 hours, lysed and extracted protein was analyzed using specific antibodies. β-actin was used as a loading control. Autophagy induction is demonstrated by an increase in the conversion of LC3I to LC3II, BECN expression and a decrease in p62 expression in the treated group as compared with the untreated. **E.** Treatment of OS cells with GCB (1μM, 48 hours), led to an increase in the formation of autophagic vacuoles (shown with red arrows) measured by transmission electron microscopy.

First, we looked at the formation of acidic vesicular organelles (AVOs) following treatment with GCB. AVO formation has been associated with autophagy induction [19]. The lysosomotropic agent acridine orange was used to measure AVO formation, as described in Materials and Methods. Acridine orange enters the acidic lysosome, becomes protonated, and emits a red fluorescence that can be measured by flow cytometry. Therefore, upon induction of autophagy, AVO formation is evidenced by bright red staining. As shown in Figure 2A, autophagy was induced in the GCB-treated OS cells, as evidenced by an increase in the formation of AVOs compared with the untreated controls. The percentage of cells with AVO formation was quantified by flow cytometry, as described in Materials and Methods. As shown in Figure 2B, an increased percentage of cells with AVO formation was observed in all three cell lines after treatment with GCB compared with untreated controls: LM7: 8.1% as compared to 1.6%; CCH-OS-D: 10% as compared to 1.7%; and K7M3: 51.4% as compared to 1.24% respectively.

We next detected LC3I to LC3II conversion using immunoblot analysis and LC3 puncta formation using fluorescent imaging analysis (Figure 2C). Treatment of LM7, CCH-OS-D, and K7M3 cells with GCB at various doses and time points resulted in increased LC3I to LC3II conversion, as demonstrated by densitometry measurement of the LC3I/LC3II ratio (Figure 2D). Treatment with GCB induced accumulation of LC3 puncta in OS cells, as demonstrated by immunocytochemistry staining. As shown in Figure 2C, a greater number of LC3 dots was observed in the cytoplasm of GCB-treated cells than in the untreated control cells, in which LC3 dots were diffuse and weak.

To further investigate whether treatment of OS cells with GCB influences autophagic flux, we used Western blot analysis to examine the expression of SQSTM1/sequestosome 1 (p62), which forms protein aggregates that are degraded by the process of autophagy [20]. Indeed, treatment of LM7, K7M3, and CCH-OS-D OS cells with GCB induced autophagic p62 degradation, as seen in Figure 2D. We also investigated whether treatment of LM7, K7M3, and CCH-OS-D cells with GCB led to an increase in the expression of BECN, an essential protein for the formation of the isolation or phagophore membrane. Western blot analysis showed an increase in BECN expression in all the three OS cell lines after treatment with GCB (Figure 2D).

Finally, we performed ultrastructural analysis using transmission electron microscopy of OS cells treated with GCB. These experiments revealed autophagy induction in the GCB-treated cells, as determined by an increase in the formation of double-membrane autophagic vacuoles (Figure 2E). In summary, using various experimental approaches, we confirmed that GCB can induce autophagy in the three OS cell lines tested.

### GCB induces autophagy in OS in vivo

**Figure 3:**
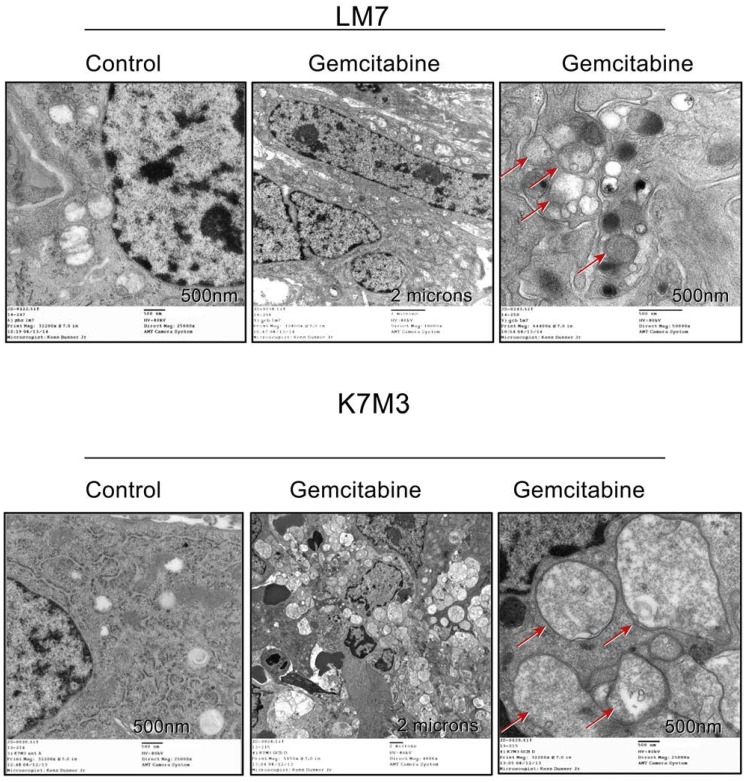
Gemcitabine induces the formation of double-membrane autophagic vacuoles in osteosarcoma lung metastases Representative electron microscopy images of lung metastases treated with gemcitabine (0.5 mg/kg), showing autophagic vesicles (arrows).

To determine whether GCB induces autophagy in OS in vivo, we analyzed lung metastases obtained from two different OS mouse models: the murine K7M3 and the human LM7 model. GCB was delivered using the aerosol route, as previously described [6]. Ultrastructural analysis of both K7M3 and LM7 lung nodules showed an increase in the number of autophagosomes and autophagolysosomes in the GCB-treated tumors compared with the phosphate-buffered saline (PBS)-treated control tumors (Figure 3), suggesting that GCB induced autophagy in OS in vivo.

### GCB-induced autophagy correlates with downregulation of the AKT/mTOR signaling pathway

After demonstrating that GCB induces autophagy in OS cells both in vivo and in vitro, we next investigated the potential implication of the AKT/mTOR signaling pathway in GCB-induced autophagy. The AKT/mTOR pathway has been described as the pathway involved in autophagy induction in other human tumors [21, 22]. Mammalian target of rapamycin (mTOR) is a well-known inhibitor of autophagy [23]. Phosphorylation of mTOR through protein kinase B, also known as AKT, results in inhibition of the process of autophagy. Thus, the AKT/mTOR signaling pathway has a role in the regulation of autophagy.

**Figure 4:**
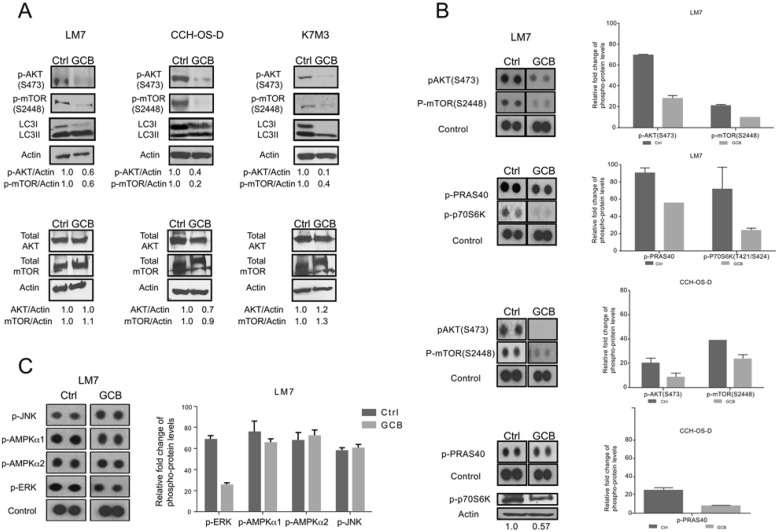
Phosphorylation of AKT and mTOR decreases after treatment with gemcitabine (GCB) **A** LM7, CCH-OS-D, and K7M3 osteosarcoma cells were treated with 1μM GCB and cell lysates were analyzed by immunoblotting with specific antibodies for AKT, mTOR, p-AKT, and p-mTOR. β-actin was used as a loading control. Densitometry was performed using ImageJ. **B.** and **C.** Following treatment with GCB, lysates were analyzed using the Human Phospho Kinase Antibody Array. The phosphorylation levels of AKT, mTOR, PRAS40, p70S6K, JNK, AMPK, and ERK were evaluated. Densitometry was performed using Image J. Values were graphed as relative fold change. Means ± standard deviation of three independent experiments are shown.

We tested the effect of GCB on AKT and mTOR phosphorylation using Western blot analysis. As shown in Figure 4A, treatment with GCB resulted in decreased AKT (S473) and mTOR (2448) phosphorylation and conversion of LC3I to LC3II in OS cells compared with their respective untreated controls. The non-phosphorylated form was not affected.

To further confirm the involvement of this signaling pathway, a high-throughput screening was performed to test the phosphorylation status of other kinases using a Human Phospho Kinase Antibody Array (R&D Systems). High-throughput screening results confirmed the inhibitory effect of GCB on p-AKT (S473) and p-mTOR (2448) in LM7 and CCH-OS-D cells (Figure 4B and 4C) as previously detected by Western blot analysis. The downstream members of the AKT/mTOR signaling pathway p70S6K and proline-rich AKT substrate of 40 kDa PRAS40 [24] were also decreased in GCB-treated OS cell lines as compared to the untreated control cells.

Induction of autophagy can also be regulated by activation of other kinases such as c-Jun N-terminal kinase (JNK), extracellular signal regulate kinase (ERK) and 5’ adenosine monophosphate-activated protein kinase (AMPK) [23]. Therefore, we also evaluated the effect of GCB on the phosphorylation status of these proteins. It was observed that p-ERK levels decreased in GCB-treated cells when compared to untreated cells. Expression levels of both p-AMPK isoforms (AMPKα1 and AMPKα2) did not change when comparing GCB treated cells to control cells. Similar results were obtained for the pJNK expression levels (Figure 4C). Taken together, these results suggest that the AKT/mTOR signaling pathway is involved in GCB-induced autophagy in OS.

### Autophagy inhibition sensitizes LM7 OS cells to GCB and decreases the sensitivity of CCH-OS-D and K7M3 OS cells to GCB

**Figure 5:**
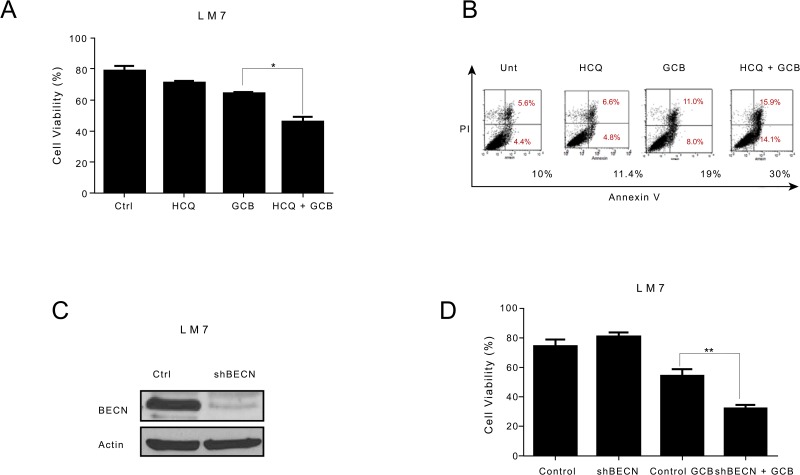
Inhibition of autophagy increases the sensitivity of LM7 osteosarcoma cells to gemcitabine (GCB)-induced cytotoxicity **A** LM7 cells were pretreated for 30 minutes with 20μM hydroxychloroquine (HCQ), 1μM GCB, or both for 48 hours. Cell viability was measured using Trypan blue exclusion assay. **B** Apoptosis was measured using propidium iodide/Annexin V staining. Cells were treated with GCB alone, HCQ alone, or HCQ combined with GCB for 48 hours. After incubation with Annexin V-FITC in a buffer containing propidium iodide, cells were analyzed using flow cytometry. **C.** After knockdown of BECN by shRNA, whole cell lysates were analyzed for BECN expression by Western blot. β-actin was used as a loading control. **D.** shRNA-BECN cells and LM7 control cells were treated with 1μM GCB for 48 hours. Cell viability was measured using Trypan blue exclusion assay. Means ± standard deviation of three independent experiments are shown. *p < 0.05 compared with control.

**Figure 6:**
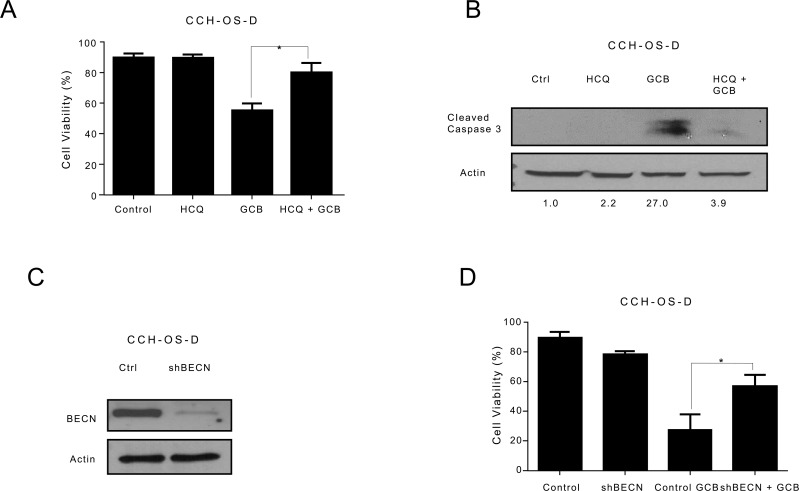
Inhibition of autophagy decreases the sensitivity of CCH-OS-D osteosarcoma cells to gemcitabine (GCB)-induced cytotoxicity **A.** CCH-OS-D cells were pretreated for 30 minutes with 20μM hydroxychloroquine (HCQ), 1μM GCB, or both for 48 hours. Cell viability was measured using Trypan blue exclusion assay. **B.** Apoptosis was evaluated using Western blot analysis with an antibody specific for cleaved caspase 3. Cells were treated with GCB alone, HCQ alone, or HCQ combined with GCB for 48 hours. After treatment, cell lysates were analyzed by Western blot. β-actin was used as a loading control. **C** After knockdown of BECN by shRNA, whole cell lysates were analyzed for BECN expression by Western blot **D.** shRNA-BECN cells and CCH-OS-D control cells were treated with 1μM GCB for 48 hours. Cell viability was measured using Trypan blue exclusion assay. Means ± standard deviation of three independent experiments are shown. *p < 0.05 compared with control.

**Figure 7:**
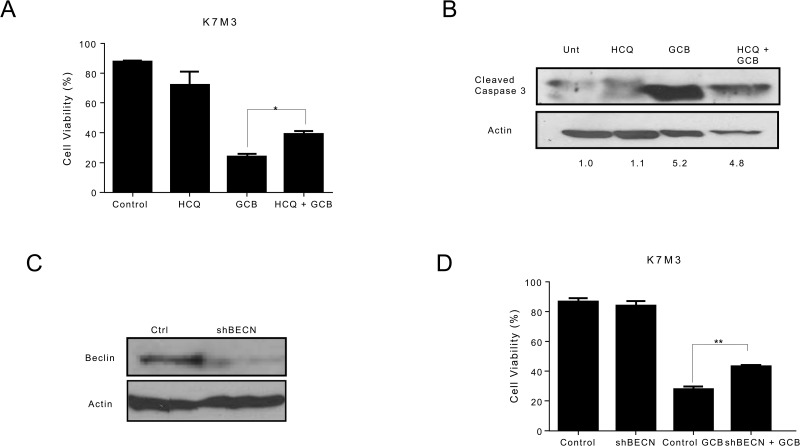
Inhibition of autophagy decreases the sensitivity of K7M3 osteosarcoma cells to gemcitabine (GCB)-induced cytotoxicity **A.** K7M3 cells were pretreated for 30 minutes with 20μM hydroxychloroquine (HCQ), 1μM GCB, or both for 48 hours. Cell viability was measured using Trypan blue exclusion assay. **B** Apoptosis was evaluated using Western blot analysis with an antibody specific for cleaved caspase 3. Cells were treated with GCB alone, HCQ alone, or HCQ combined with GCB for 48 hours. After treatment, cell lysates were analyzed using Western blot. β-actin was used as a loading control. **C** After knockdown of BECN by shRNA, whole cell lysates were analyzed for BECN expression by Western blot. **D** shRNA-BECN cells and K7M3 control cells were treated with 1μM GCB for 48 hours. Cell viabilitiy was measured using Trypan blue exclusion assay. Means ± standard deviation of three independent experiments are shown. *p < 0.05 compared with control.

To determine whether GCB-induced autophagy contributes to OS cell survival or cell death, we inhibited autophagy using two approaches. First we used HCQ, a pharmacologic agent known to inhibit autophagy by blocking the fusion between autophagosomes and lysosomes, thus acting at the late stage of the autophagy process [24, 25]. HCQ has been shown to enhance the efficacy of some chemotherapeutic agents when used in combination for the treatment of other malignancies [10, 26]. Treatment of LM7, CCH-OS-D, and K7M3 OS cells with 20μM HCQ alone did not affect cell viability (Figures 5A-7A). Pretreatment of LM7 OS cells with HCQ increased cell sensitivity to GCB as demonstrated by a decrease in cell viability. Furthermore, inhibition of autophagy in GCB-treated LM7 OS cells demonstrated enhanced propidium iodide/Annexin V staining suggesting that combination therapy enhanced cell death (Figure 5B).

In contrast, combination treatment with HCQ and GCB in CCH-OS-D and K7M3 OS cells significantly decreased cell sensitivity to GCB (Figures 6A and 7A). Moreover, combination therapy in these cells also led to a decrease in cleaved caspase 3 (Figures 6B and 7B), suggesting that induction of autophagy in these cells contributed to cell death.

Pharmacologic inhibition can be limited due to its potential effect on other cellular processes in addition to autophagy. To further validate the importance of GCB-induced autophagy in OS response or resistance, we genetically inactivated this process using shRNA to knock down the expression of Beclin (BECN), an essential autophagy protein. BECN shRNA transfection led to a significant decrease in BECN protein expression, as shown in Figure 5C. Knockdown of BECN expression in LM7 OS cells rendered them significantly more sensitive to GCB-induced cell death (Figure 5D). In contrast, knockdown of BECN expression in CCH-OS-D cells significantly decreased sensitivity to GCB-induced cell death. (Figure 6D). The same effect was seen in K7M3 cells infected with shBECN after treatment with GCB (Figure 7D). These results indicate that GCB-induced autophagy decreases drug therapeutic response in LM7 OS cells but increases the therapeutic effect in CCH-OS-D and K7M3 OS cells. In addition, knock down of ATG5 expression in CCH-OS-D and K7M3 cells also decreased sensitivity to GCB (data not shown).

We demonstrated that GCB-induced autophagy can either promote OS tumorigenicity or lead to cell death. However, what determines whether induction of autophagy will lead to survival or death remains unknown.

### Chemotherapy-induced phosphorylated HSP27 expression correlates to whether inhibition of autophagy sensitizes OS cells to therapy

Thorough analyses of some of the additional kinases expressed in the phosphokinase array after treatment with GCB demonstrated striking differences in the expression of p-HSP27 among the cell lines tested. Heat shock proteins are known to be increased when cells are exposed to elevated temperatures and other types of stress. In particular, the expression of HSP27 has been associated with poor prognosis in OS [27, 28]. Furthermore, high levels of p-HSP27 have been shown to be involved in the resistance of pancreatic cancer cells to GCB [29, 30].

**Figure 8:**
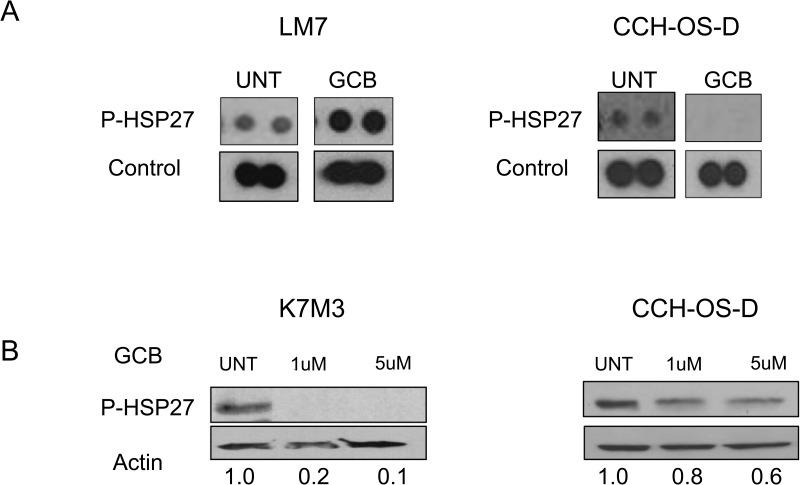
Expression of phosphorylated heat shock protein 27 (p-HSP27) varies in different types of osteosarcoma cells after treatment with GCB **A** LM7 and CCH-OS-D cells were treated with 1μM gemcitabine (GCB) for 48 hours. Cell content and expression of p-HSP27 was analyzed using the Human Phospho Kinase Antibody Array. **B.** K7M3 and CCH-OS-D cells were treated with the indicated doses of GCB. Cells were collected and lysed with RIPA buffer. Western blot analysis was performed using an antibody specific for p-HSP27 (Ser78/82). β-actin was used as a loading control.

We observed a difference in the expression of p-HSP27 between LM7 and CCH-OS-D cells upon treatment with GCB (Figure 8A). Increased levels of p-HSP27 were observed in GCB-treated LM7 cells, in which blocking autophagy led to enhanced cell death (Figure 8A), whereas decreased levels of p-HSP27 were observed in CCH-OS-D GCB-treated OS cells, in which blocking autophagy led to cell survival (Figure 8A). These findings suggest that induction of p-HSP27 influences the effect of autophagy inhibition following GCB treatment. As shown in Figures 5 and 8, blocking autophagy in LM7 cells, in which p-HSP27 was increased following treatment with GCB, resulted in enhanced drug sensitivity, whereas blocking autophagy in CCH-OS-D cells, in which p-HSP27 was decreased following treatment with GCB, resulted in reduced drug sensitivity (Figures 6 and 8).

**Figure 9:**
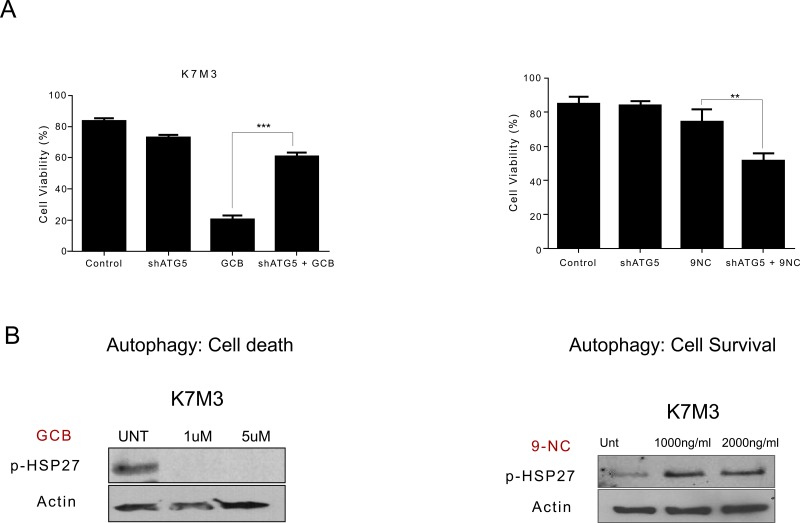
Variation in phosphorylated heat shock protein 27 (HSP27) expression after treatment with gemcitabine (GCB) or 9-nitrocamptothecin (CPT) correlates to whether blocking autophagy will lead to survival or death in K7M3 osteosarcoma cells **A.** shATG5-K7M3 cells were treated with GCB or CPT and cell viability was assessed using Trypan blue exclussion assay. Mean ± standard deviation of three independent experiments are shown. *p < 0.05 compared with control. shATG5-K7M3 cells were less sensitive to GCB but more sensitive to CPT. **B** K7M3 cells were lysed with RIPA buffer and subjected to Western blot analysis with the antibody specific for p-HSP27. β-actin was used as a loading control. p-Hsp27 decreases in the K7M3 GCB treated cells and increases in the K7M3 9-NC treated cells.

To determine if this relationship is drug-specific, we assessed the expression of p-HSP27 in K7M3 cells treated with CPT. We have previously demonstrated that autophagy inhibition increases K7M3 sensitivity to CPT-induced cell death [12] as opposed to the current observation in which autophagy inhibition decreases K7M3 sensitivity to GCB-induced cell death. We observed increased expression of p-HSP27 after treatment with CPT (Figure 9B) as opposed to a decreased expression after treatment with GCB (Figure 8B) in the K7M3 cells, suggesting that the effect on p-HSP27 is drug specific and that pHSP27 could serve as a potential biomarker to predict the role of chemotherapy-induced autophagy in OS. Further studies are underway to confirm the role of p-HSP27 in the context of chemotherapy-induced autophagy in OS.

### HSP27 expression varies in human OS primary tumors and lung metastases

**Figure 10:**
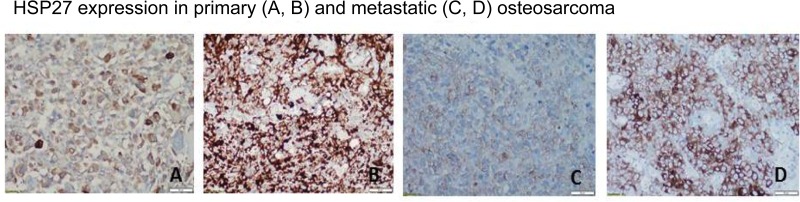
Expression of heat shock protein 27 (HSP27) varies in human osteosarcoma primary tumors and lung metastases Representative images of HSP27 expression in patient samples are shown. Using an institutional review board-approved established osteosarcoma tissue microarray, we performed immunohistochemistry staining for HSP27 using an HSP27-specific antibody. HSP27 expression varied between 0% and 100% in both primary resection specimens (obtained following treatment with neoadjuvant chemotherapy in most cases) and resected pulmonary metastasis specimens. HSP27 was expressed (defined as >10% staining in tumor tissue) in 81 of 132 primary resection specimens (61%) and 50 of 64 osteosarcoma pulmonary metastasis specimens (78%).

We preliminarily tested HSP27 expression in OS samples from patients to identify potential differences in expression and determine how these differences could affect autophagy and response to chemotherapy. We used an institutional review board-approved, established OS tissue microarray that contains samples of primary tumors and lung metastases. Using the grading system described in Materials and Methods, we found that, according to immunohistochemistry staining, HSP27 expression varied between 0% and 100% in both the primary specimens and the lung metastases obtained after initial chemotherapy. HSP27 expression was positive in 81 of 132 OS primary tumor samples (61%) and 50 of 64 OS pulmonary metastasis samples (78%). Representative images are shown in Figure 10. Further studies are necessary to determine whether the differences in HSP27 expression are correlated with autophagy and response to chemotherapy.

## DISCUSSION

In the present study, we demonstrated that GCB induces autophagy in different OS cells, as shown by an increase in AVO formation, LC3I to LC3II conversion, and BECN expression, in addition to a decrease in p62 expression (Figure 2). Furthermore, autophagy induction was evidenced by an increase in the formation of autophagic vesicles not only in the OS cell lines but also in OS lung metastases from mice (Figure 3). We also confirmed inhibition of the AKT/mTOR signaling pathway and downstream components PRAS40, and p70S6K upon GCB-induced autophagy (Figures 4 and 5). This mechanism of autophagy induction has previously been described in other diseases [21, 22, 31].

Inhibition of autophagy, by the pharmacologic autophagy inhibitor HCQ, a specific inhibitor of the autophagosome-lysosome fusion or shRNA knockdown of BECN expression, significantly enhanced the sensitivity of LM7 OS cells to GCB, as demonstrated by a decrease in cell viability (Figure 5). These results are consistent with what has been found in several other studies where chemotherapy promotes cytoprotective autophagy and that chemotherapy sensitization can be mediated through pharmacologic or genetic inhibition of autophagy. However, using the same approach, we found that inhibition of autophagy in CCH-OS-D and K7M3 OS cells significantly decreased the sensitivity of these cells to GCB, as demonstrated by an increase in cell viability and a decrease in cleaved caspase 3 (Figures 6 and 7), confirming the opposing effect of GCB-induced autophagy in the CCH-OS-D and K7M3 OS cells as compared to the LM7 cells. These results are consistent with our published work where we demonstrated that treatment of DLM8 and K7M3 OS cells with CPT, a different chemotherapeutic agent, also induced autophagy and that blocking autophagy in these cells led to survival in the DLM8 cells and death in the K7M3 cells [12]. Taken together, our results clearly demonstrate the dual role of autophagy, both cytoprotective and cytotoxic, in OS. However, what determines whether chemotherapy-induced autophagy will lead to survival or death remains a key question yet to be answered.

In the present study, we showed for the first time that expression of p-HSP27 is associated with whether chemotherapy-induced autophagy will lead to survival or death. We found that induction of p-HSP27 following exposure to 9-NC or GCB correlates with the role of autophagy in drug sensitivity. Heat shock proteins are a class of functionally related proteins whose expression is increased under stressful conditions. HSPs protect cells from stress-associated injury, are overexpressed in many malignancies, and are implicated in tumor cell proliferation, differentiation, invasion, and metastasis [19, 20]. In particular, HSP27 (also known as HSPB1) is a stress-activated molecular chaperone that acts as an antioxidant and as an apoptosis inhibitor, protecting cells from chemotherapy-induced cell death [32]. Overexpression of HSP27 was associated with poor prognosis in gastric, liver, prostate, breast cancer and OS [32, 33]. HSP27 has also been shown to be a predictive biomarker of chemotherapy resistance [33]. In OS, HSP27 was overexpressed in 22-24% of patients at baseline, increasing to 33-37% at resection following neoadjuvant chemotherapy [27, 32]. Overexpression was associated with lower overall survival [27].

Our studies demonstrate p-HSP27 as a potential biomarker to whether chemotherapy-induced autophagy will lead to survival or death. Monitoring expression levels of HSP27 in patients with OS may help identify those who will benefit from a combination therapy that includes an autophagy inhibitor. Here we used representative samples of OS primary tumors and pulmonary metastases and confirmed various expression levels of HSP27 among the different samples analyzed (Figure 10). Further studies are needed to correlate Hsp27 expression and determine the exact relationship between HSP27 expression and autophagy, disease status (relapsed/recurrent disease), as well as response to chemotherapy.

Our findings provide significant insight into the role of autophagy in OS and highlight the importance of understanding the implications of autophagy in OS response to chemotherapy. Recently, enthusiasm has developed around the idea of autophagy modulation as a novel strategy for the treatment of multiple types of cancer. Clinical trials are currently ongoing to investigate the role of combination treatment with chemotherapy and autophagy inhibitors such as HCQ. However, data remain inconclusive. The autophagy paradox remains unanswered. Whether blocking chemotherapy-induced autophagy translates to a beneficial or detrimental outcome is still under active investigation. Caution should be taken before attempting combination therapy with autophagy modulators and chemotherapeutic drugs. The role of autophagy, cytoprotective or cytotoxic, should be established for the specific intended target or treatment combination. A deeper understanding of the biologic underpinning that defines the role of drug-induced autophagy in OS and other cancers is critical for the implementation of more effective and rational combination therapies. Our ongoing studies may unveil the specific function of p-HSP27 in defining the role of chemotherapy-induced autophagy in OS. This will help determine the predictive value of HSP27 as a potential biomarker to define the role of chemotherapy-induced autophagy in OS.

## MATERIALS AND METHODS

### OS cell lines and cell culture

K7M3, LM7, and CCH-OS-D metastatic OS cell lines were cultured in Dulbecco's modified Eagle medium containing 10% fetal bovine serum supplemented with antibiotic and nonessential amino acids. Cells were maintained in a humidified incubator with 5% CO_2_ at 37°C. Each cell line was tested for mycoplasma contamination.

### Reagents and antibodies

GCB was obtained from Eli Lilly and dissolved in PBS for a final concentration of 38 mg/ml. HCQ was obtained from Sigma Aldrich and dissolved in water to a final concentration of 10mM. Acridine orange (A8097) was obtained from Sigma Aldrich. RIPA lysis buffer (sc-24948) was obtained from Santa Cruz Biotechnology. Microtubule-associated protein 1 light chain LC3B (NB600-1384) was purchased from Novus Biologicals. Beclin (sc-11427) and HSP27 (sc-13132) were obtained from Santa Cruz Biotechnology. SQSTM1/p62 (5114), AKT (4685), p-AKT (9271), mTOR (2983), p-mTOR (Ser2448; 2971), p-p70S6K (Thr389; 9205), and cleaved caspase 3 (Asp175; 9691) were obtained from Cell Signaling Technology. p-HSP27 (MAB23141) was purchased from R&D Systems.

### Flow cytometry

Apoptosis was assessed by flow cytometric analysis of Annexin V. LM7 cells were treated with 20μM HCQ or 1μM GCB or combination of HCQ and GCB. Floating and adherent cells were collected after 72 hours of treatment with GCB, fixed, and stained using the Annexin V-FITC apoptosis detection kit (Calbiochem, #PF032). The samples were analyzed using a FACS Calibur flow cytometer (Becton-Dickinson).

### Cell viability

Cells were seeded in 12-well plates with approximately 0.5 × 10^5^ cells per well and allowed to attach overnight at 37°C, 5% CO_2_. Cells were then treated with GCB or GCB coupled with HCQ; cells with no treatment served as controls. Cells were trypsinized and viability was read using a cell counter (Vi-cell, Beckman Coulter). All experiments were performed three times. The incubation time and drug concentrations are indicated in each figure.

### Acridine orange staining

K7M3, LM7, and CCH-OS-D cells were treated with 1μM GCB for 48 hours. Following treatment with GCB, cell culture media was removed and cells were stained with 1 mg/ml acridine orange and incubated for 15 minutes at 37°C. After incubation, cells were washed twice with PBS and analyzed using the FL3 channel on a flow cytometer using Cell-Quest software (Becton-Dickinson). Alternatively, cells were examined under a fluorescent microscope. The autophagic lysosomes appeared as orange vesicles in the cytoplasm of the cells, whereas nuclei stained green.

### Human phospho-kinase Antibody Array Kit

LM7 and CCH-OS-D OS cells were treated with GCB during 48 or 72 hours. Untreated cells were used as a control. The cells were collected and lysed using the Lysis buffer provided by the manufacturer, and protein concentration was determined using the Bradford Protein Assay from Bio-Rad. After blocking, the membranes were incubated with 400ug of protein overnight at 4°C. The membranes were washed and incubated with detection antibody cocktails. HRP-conjugated streptavidin antibodies and chemniluminescent detection reagents were used to visualize the protein.

### Western blot analysis

After treatment, whole cell lysates were prepared by lysing the cells with RIPA lysis buffer for 30 minutes and centrifuging at 10,000 g at 4°C. Supernatants were collected and protein concentration was determined using the Bio-Rad DC protein assay kit (500-0113-0115). Equal amounts of protein were resolved in SDS-polyacrylamide gels (SDS-PAGE) and transferred onto a nitrocellulose membrane. Membranes were blocked in 5% milk or 5% bovine serum albumin for 1 hour and then incubated with primary antibody against AKT (1:1000), p-AKT (1:1000), Beclin (1:1000), cleaved caspase 3 (1:1000), LC3 (1:1000), p-mTOR (1:1000), mTOR (1:1000), p62 (1:1000), or p-HSP27 (1:1000). After overnight incubation with primary antibodies, membranes were washed and incubated with anti-mouse (1:2000) horseradish peroxidase linked whole antibody (from sheep, NA931V; GE Healthcare) or anti-rabbit (1:2000) horseradish peroxidase linked whole antibody (from donkey, NA934V; GE Healthcare) as a secondary antibody. Signal was detected using ECL reagents (GE Healthcare Life Science). β-actin was used as a loading control.

### RNA interference

Lentiviral Beclin shRNA (ThermoFisher) was used to knock down BECN protein expression. Control cells were infected with lentivirus containing empty shRNA vector. Briefly, 293T cells were transfected with the plasmid at a concentration of 7 μg/ml target plasmid, 5 μg/ml psPAX2, and 4 μg/ml pMD2G. After 48 hours, the supernatant was collected and used for infection. For infection, 2 ml of supernatant containing lentivirus was added to each well of a 6-well plate containing 1 × 105 cells. Cells were incubated with lentivirus for 12 hours and next transferred to a 75-mm flask. Confirmation of protein knockdown was determined using Western blot analysis.

### Confocal microscopy for LC3

K7M3, CCH-OS-D, and LM7 cells were grown on glass coverslips and treated with 1μM GCB. After 48 hours of treatment, cells were fixed with 4% paraformaldehyde at room temperature for 15 minutes. Following three washes with PBS, cells were incubated with anti-LC3 antibody (1:100). After overnight incubation, cells were incubated with Alexa 488 mouse anti-rabbit antibody (Life Technologies) at 1:400 dilution for 1 hour and immediately analyzed by confocal microscopy. Green punctate staining depicted LC3 staining.

### Electron microscopy

K7M3, LM7, and CCH-OS-D cells were treated with 1μM GCB for 48 hours. Next, samples were fixed with a solution of 3% glutaraldehyde plus 2% paraformaldehyde in 0.1M cacodylate buffer, with a pH of 7.3. After fixation, the samples were washed in 0.1M cacodylate buffer and treated with 0.1% Millipore-filtered buffered tannic acid. The samples were then postfixed with 1% buffered osmium tetroxide for 30 minutes and stained with 1% Millipore-filtered uranyl acetate. Samples were washed several times in water, then dehydrated in increasing concentrations of ethanol, after which they were infiltrated and embedded in LX-112 medium. The samples were polymerized in an oven at 60°C for 2 days. Ultrathin sections were cut using a Leica Ultracut microtome (Leica), and these sections were stained with uranyl acetate and lead citrate using a Leica EM stainer and examined using a JEM 1010 transmission electron microscope (JEOL, USA, Inc.) at an accelerating voltage of 80 kV. Digital images were obtained using the AMT Imaging System (Advanced Microscopy Techniques Corp).

### Tissue Microarray

Four micron thick unstained slides were prepared from tissue microarrays containing formalin-fixed paraffin-embedded decalcified human osteosarcoma specimens. The tissue microarrays were constructed from 116 biopsies, 184 primary resection specimens, and 94 metastases and derived from 265 patients. Immunohistochemistry was performed using anti-human HSP27 monoclonal antibody (1:1000, Thermo Fischer Scientific, clone MA3-15) for 1 hr at room temperature following antigen retrieval with 10 mM citrate buffer (pH 6.0) and microwave oven at 3 minutes. Immunoreactivity was scored for intensity (weak, moderate or strong) and extent (percentage of tumor staining).

### Statistical analysis

Data were expressed as means of three independent experiments. Experimental data were analyzed using Graphpad PRISM 6.0 software. p < 0.05 was considered statistically significant.

### Image densitometry

Gel image densitometry was obtained using NIH ImageJ software.

## Abbreviations

OS: osteosarcoma
GCB: gemcitabine
9-nitrocamptothecin: CPT
AVOs: acidic vesicular organelles
mTOR: mammalian target of rapamycin
HCQ: hydroxychloroquine
HSP27: heat shock protein 27
PBS: phosphate-buffered saline.
